# The Role of the Gut Microbiota in Complications among Hemodialysis Patients

**DOI:** 10.3390/microorganisms12091878

**Published:** 2024-09-12

**Authors:** Junxia Du, Xiaolin Zhao, Xiaonan Ding, Qiuxia Han, Yingjie Duan, Qinqin Ren, Haoran Wang, Chenwen Song, Xiaochen Wang, Dong Zhang, Hanyu Zhu

**Affiliations:** 1 Department of Nephrology, First Medical Center of Chinese People’s Liberation Army General Hospital, Nephrology Institute of the Chinese People’s Liberation Army, National Key Laboratory of Kidney Diseases, National Clinical Research Center for Kidney Diseases, Beijing Key Laboratory of Kidney Disease Research, Beijing 100853, China; junxiadu123@163.com (J.D.); zhaoxiaolin301@126.com (X.Z.);; 2 Medical School of Chinese People’s Liberation Army, Beijing 100853, China; 3 Department of Nephrology, Beijing Chao-Yang Hospital, Capital Medical University, Beijing 100020, China; qiuxiahanamy@163.com

**Keywords:** gut microbiota, hemodialysis, complications, uremic toxins

## Abstract

The composition of the gut microbiota varies among end-stage renal disease (ESRD) patients on the basis of their mode of renal replacement therapy (RRT), with notably more pronounced dysbiosis occurring in those undergoing hemodialysis (HD). Interventions such as dialysis catheters, unstable hemodynamics, strict dietary restrictions, and pharmacotherapy significantly alter the intestinal microenvironment, thus disrupting the gut microbiota composition in HD patients. The gut microbiota may influence HD-related complications, including cardiovascular disease (CVD), infections, anemia, and malnutrition, through mechanisms such as bacterial translocation, immune regulation, and the production of gut microbial metabolites, thereby affecting both the quality of life and the prognosis of patients. This review focuses on alterations in the gut microbiota and its metabolites in HD patients. Additionally, understanding the impact of the gut microbiota on the complications of HD could provide insights into the development of novel treatment strategies to prevent or alleviate complications in HD patients.

## 1. Introduction

The gut microbiota refers to the extensive microbial communities present in the human gut, encompassing bacteria, fungi, and viruses. Advances in metagenomics, 16S rRNA sequencing, and metabolomics have provided a more comprehensive understanding of the gut microbiota. In healthy individuals, the gut microbiota is distributed in specific quantities and proportions in different regions of the gut and has a significant impact on various aspects of human health [1]. First, in terms of nutrient metabolism, the gut microbiota synthesizes a variety of vitamins (such as those from groups K and B) and can degrade indigestible food components such as plant polysaccharides and oxalate. Furthermore, the gut microbiota supports normal colonic epithelial cell function by producing short-chain fatty acids (SCFAs), thereby promoting the stability of the intestinal mucosal barrier. Additionally, immune regulation represents another pivotal role played by the gut microbiota. It can enhance host immune function and modulate responses to infections and autoimmune diseases through either the inhibition or promotion of specific immune reactions. Finally, complex interrelationships exist between the gut microbiota and other organs such as the heart, brain, and kidneys [2,3,4].

Chronic kidney disease (CKD) is a global health challenge characterized by progressive decline in kidney function and is influenced by a multitude of factors [5]. In addition to established risk factors such as hypertension and diabetes, increasing evidence indicates that the gut microbiota plays a pivotal role in CKD. The link between CKD and the gut microbiota can manifest through the gut–kidney axis. In individuals with CKD, uremic toxins accumulate due to declining kidney function, resulting in a uremic environment [6]. Under these circumstances, dysbiosis of the gut microbiota becomes prevalent. Furthermore, dysbiosis of the gut microbiota compromises intestinal barrier integrity, resulting in increased translocation of endotoxin and uremic toxins from the gut, activation of inflammatory responses, and the onset of various complications [7,8].

Hemodialysis (HD) is the most common renal replacement therapy (RRT) for patients with end-stage renal disease (ESRD) [9]. With the increasing number of patients with CKD, the demand for HD has also significantly increased. The number of patients receiving RRT worldwide is predicted to reach 5.439 million by 2030 [10]. In patients receiving HD treatment, a variety of complications, including cardiovascular disease (CVD), infections, anemia, malnutrition, cognitive decline, and psychological health problems, often occur, seriously affecting their quality of life [11,12]. Although considerable progress in dialysis technology has been made and waste removal has improved, there are still limitations in dealing with uremic toxins produced by the gut microbiota. This may lead to challenges for ESRD patients even after receiving dialysis treatment, as they still face the accumulation of gut-derived uremic toxins. 

This review discusses the changes in the gut microbiota and its metabolites in HD patients, as well as the effects of the gut microbiota on HD complications, to provide insights for treatment and diagnosis from the perspective of the gut microbiota.

## 2. The Gut Microbiota and HD

There are 100 trillion microbes in our intestinal tract [13]. In healthy people, Firmicutes and Bacteroidetes account for approximately 90% of the microbiota, followed by Actinobacteria, Proteobacteria, Fusobacteria, and Verrucomicrobia [14]. The gut microbiota of ESRD patients (predialysis and postdialysis) undergoes profound changes [15]. This is related to the rapid decline in renal function in ESRD patients, the accumulation of toxins in the body, the large amount of urea circulating into the gut lumen, and the stimulation of the overproduction of urease-containing bacteria [16,17]. Special dietary restrictions (reduced intake of fruits, vegetables, and dairy products), comorbidities (diabetes, hypertension, etc.), and medications (phosphate binders, iron, and antibiotics) can also lead to gut microbiota dysbiosis [18,19,20,21]. In HD, some waste products in the blood are removed through the semipermeable membrane, which means that there are vascular access interventions (arteriovenous fistula/graft or venous catheter), inadequate dialysis, and low immunity, which may further aggravate gut microbiota dysbiosis [22,23]. 

The results of studies concerning alterations in the gut microbiota in HD patients are summarized in Table 1. Proteobacteria and Firmicutes were increased in HD patients compared with healthy controls [18]. This observation is consistent with recent findings reported by Wu et al. [24]. However, some investigators have shown that the abundance of Firmicutes is significantly reduced in HD patients [23]. The abundance of Bacteroidetes shows different trends in adult and pediatric HD patients [24,25]. The differences in these research results may be due to variations in the subjects’ age, genetic history, diet, lifestyle, and dialysis adequacy. Owing to the lack of comparative studies before and after dialysis in patients with ESRD, we cannot accurately determine the specific causes of the microbiome changes mentioned above. These changes may be caused by dialysis treatment itself, by ESRD itself, or even by the combined effects of both. To gain a deeper understanding of these changes, Lou et al. specifically selected ESRD patients who had not yet started dialysis, peritoneal dialysis (PD) patients, and HD patients for a detailed comparative analysis, and their findings indicated that HD treatment had a more significant effect on the gut microbiota of ESRD patients. Their study revealed that HD significantly increased the proportion of beneficial bacteria in the microbial community but also stimulated the growth and reproduction of certain potentially pathogenic bacteria, which may introduce new risks to the stability of ecosystems [26]. At the phylum level, HD patients presented the lowest levels of Bacteroidetes [26]. At the genus level, there was a decrease in the abundance of *Prevotella* and *Paraprevotella* and an increase in the abundance of *Akkermansia*, *Coprococcus*, *Acinetobacter*, *Proteus*, and *Pseudomonas* [26]. *Prevotella* and *Paraprevotella*, two anaerobic Gram-negative rods associated with infections of the gastrointestinal, respiratory, and urinary tracts, are rich in peptidases [27], which degrade proteins and produce large amounts of ammonia [28]. A high concentration of ammonia destroys intestinal epithelial tight junctions (TJs), leading to intestinal mucosal injury [29]. *Akkermansia* and *Coprococcus* are associated with the production of SCFAs. *Akkermansia* is a promising probiotic resident in the mucus layer that reduces inflammation and improves host metabolism [30]. Studies have shown that increasing the abundance of *Akkermansia* may reduce the chronic inflammatory state of CKD patients [31]. *Acinetobacter* is an important opportunistic pathogen in hospitalized patients [32]. *Proteus*, family *Enterobacteriaceae*, is a common commensal bacterium in the gastrointestinal tract that secretes virulence factors such as the protease ZapA and is therefore potentially pathogenic [33]. The abundance of *Pseudomonas* was positively correlated with plasma tryptophan levels [34]. Tryptophan is an essential amino acid that can be fermented by gut microbiota into various bioactive metabolites, of which indoles and their derivatives are the most notable group. Some indole derivatives are believed to promote the formation of uremic toxins, thereby aggravating the patient’s condition [35].

The gut and kidney are linked through two pathways of metabolism and immunity, known as the gut–kidney axis [7]. The metabolic-dependent pathways are mediated mainly by metabolites produced by the gut microbiota, which can regulate the physiological functions of the host. On the one hand, the production of beneficial SCFAs in HD patients is reduced, which may be related to the reduction in the levels of SCFA-producing bacteria such as *Lactobacillus*, *Prevotellaceae*, and *Ruminococcus* [17,36]. SCFAs, including acetate, propionate, and butyrate, serve as major sources of nutrients for colon cells [37] and can be absorbed into the bloodstream through the intestinal lumen for transport to distant organs, such as the heart, kidney, muscle, and adipose tissue, where they provide a source of energy for host metabolism [38,39,40]. In addition, SCFAs also have anti-inflammatory effects [41]. Among them, butyrate has potent anti-inflammatory effects and reduces the levels of the inflammatory factors tumor necrosis factor-α (TNF-α) and interleukin-6 (IL-6) by inhibiting the activation of the nuclear factor kappa B (NF-κB) cell signaling pathway [42]. On the other hand, the levels of uremic toxins such as indoxyl sulfate (IS) and p-cresyl sulfate (pCS) in the plasma of HD patients are elevated [43], which is linked to the proliferation of bacteria that produce urease, uricase, p-cresol, and indoles (specifically, *Clostriadiaceae* and *Enterobacteriaceae*) [17]. IS is derived from a small molecule produced by dietary tryptophan metabolism. Tryptophan is metabolized to indole by colonic bacteria such as *Escherichia coli*; then, indole enters the liver from the portal vein and is further oxidized and sulfated to IS [4]. pCS is generated as a product of the metabolism of tyrosine and phenylalanine. Tyrosine and phenylalanine are metabolized by anaerobic bacteria in the colon into p-cresol. p-Cresol is further converted in the liver to pCS [4]. When IS and pCS enter the circulation, these solutes bind to plasma albumin at a binding rate of up to 90% [44]. As renal function declines in ESRD patients, IS and pCS accumulate in the blood, with IS and pCS concentrations in predialysis patients being 116 and 41 times higher than those in normal subjects, respectively [43]. Owing to the close binding of IS and pCS to albumin, the dialysis clearance rates of IS and pCS are only 0.21 times and 0.39 times greater than normal kidney clearance, whereas the clearance rates of urea and creatinine are 4.2 times and 1.3 times greater, respectively [43]. Furthermore, there are elevated levels of the gut-derived microbial metabolite trimethylamine *N*-oxide (TMAO). TMAO is a small-molecule, water-soluble poison metabolized by quaternary amines (choline and L-carnitine). Quaternary amine metabolism produces trimethylamine (TMA), which is converted to TMAO by flavin-containing monooxygenase 3 (FMO3) [45]. The plasma TMAO concentration in HD patients is more than 20 times greater than that in patients with normal kidney function [46,47]. This means that the level of uremic toxins is still high in HD patients, which may cause renal and cardiovascular toxicity and have a negative impact on the prognosis of patients [48].

**Figure 1:**
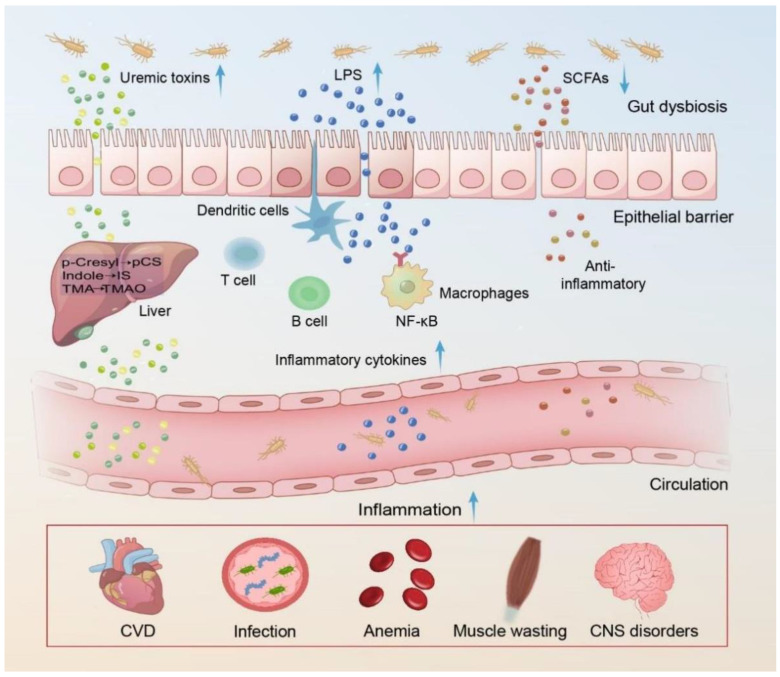
Gut dysbiosis and the accumulation of gut-derived uremic toxins in hemodialysis (HD) patients lead to systemic damage, which involves cardiovascular disease (CVD), infection, anemia, muscle wasting, and central nervous system (CNS) disorders.

Another important pathway between the gut and kidney involves immune-mediated signaling [7]. As kidney function decreases, the immune system function of CKD patients gradually deteriorates, which can lead to a chronic inflammatory state [49]. Recent evidence suggests that breakdown of the intestinal barrier, which leads to the translocation of bacteria, endotoxins, and uremic toxins from the gut to the bloodstream, is also an important factor in chronic inflammation [20]. The intestinal physical barrier is composed of a continuous layer of enterocyte cells scattered with special cell types (goblet cells, enteroendocrine cells, and Paneth cells) and sealed by intercellular junctional complexes (known as tight junctions [TJs]) [50,51]. Vaziri et al. reported a significantly decreased expression of TJ proteins in the colonic mucosa of uremic rats [52]. SCFAs play an important role in providing energy to colon cells and help maintain normal colonic mucosal barrier function. When SCFA production decreases, damage to the colonic mucosal barrier occurs, increasing intestinal permeability [53]. In addition, HD-related factors (such as the dialysis process and frequency, ultrafiltration, and intradialytic exercise) and non-HD related factors (uremia and medication) can both alter the intestinal barrier and increase intestinal permeability [53]. In this case, bacteria, endotoxins, and gut-derived uremic toxins are more likely to enter the bloodstream through the intestinal wall, inducing or aggravating inflammation [20]. A study revealed that among 30 ESRD patients, bacterial DNA was detected in the blood of 6 individuals, indicating the presence of bacterial infections or overgrowth in this subset of patients [54]. Furthermore, all observed genera (*Klebsiella* spp., *Proteus* spp., *Escherichia* spp., *Enterobacter* spp., and *Pseudomonas* spp.) in the blood of the ESRD patients exhibited overgrowth in the gut, suggesting potential dysbiosis and translocation of the gut microbiota. Additionally, the plasma levels of D-lactate, high-sensitivity C-reactive protein (hs-CRP), and IL-6 were significantly elevated in the patients with detectable bacterial DNA compared to those without it. These findings suggest that bacterial translocation occurs in patients with ESRD and is associated with microinflammation [54]. Lipopolysaccharide (LPS) is a complex biological molecule that is found in the cell walls of Gram-negative bacteria *Escherichia coli*. LPS has strong immunological activity and can bind to Toll-like receptors (TLRs) to activate NF-κB and increase the production of inflammatory cytokines, such as IL-1, IL-6, and IL-18 [55,56,57]. The gut-derived uremic toxins (IS and pCS) also play a significant role in promoting inflammation [58]. Experimental evidence has demonstrated that IS and pCS can induce leukocyte adhesion and extravasation, thereby exerting pro-inflammatory effects [59]. Additionally, they are capable of inducing the generation of reactive oxygen species (ROS), further activating the NF-κB pathway and leading to the production of pro-inflammatory cytokines [60,61]. In HD patients, studies have revealed elevated levels of inflammatory markers such as CRP and IL-6 [62]. The increase in these indicators may be associated with internal immune responses and metabolic disorders. Specifically, the level of IL-6 is positively correlated with IS and pCS, indicating a potential link between them [62]. The dysregulation of gut-microbiota-mediated host inflammatory activity influences the occurrence of complications in HD patients, consequently impacting their prognosis (Figure 1).

## 3. The Gut Microbiota and CVD

HD patients have a high mortality rate from CVD [63]. Research has revealed that the relative risk of CVD mortality in HD patients is 20 times greater than that in the general population [64]. This is mainly because HD patients are usually accompanied by a series of metabolic disorders, including hypertension, hyperglycemia, and hyperlipidemia. In addition, the chronic inflammatory state, oxidative stress, and uremic toxins, among other nontraditional risk factors, play key roles in the development of CVD in HD patients [65]. After HD treatment, the increase in intestinal permeability leads to the translocation of the gut microbiota and metabolites into the bloodstream, resulting in an increase in inflammatory cytokines. Systemic inflammation can promote the development and progression of CVD [23,66].

Several studies have revealed a significant correlation between the gut microbiota composition and CVD incidence. Among them, Sumida et al. studied the composition of circulating microorganisms in HD patients who died from cardiovascular events and reported that the abundance of Actinobacteria increased, whereas that of Proteobacteria decreased [67]. Similar findings have also been documented in comparative studies involving cohorts of healthy individuals and those diagnosed with CVD [68]. The proportions of the Actinobacteria and Proteobacteria phyla are significantly correlated with the levels of nuclear factor erythroid 2-related factor 2 (Nrf2) in the blood [67]. Nrf2 is a key regulator of the antioxidant response and plays a crucial role in immune regulation. Dysregulation of Nrf2 activation is associated with the occurrence and progression of CVD [69]. In ESRD patients, analysis of CVD mortality data revealed a decrease in *Bacteroides* and *Phascolarctobacterium*, suggesting that these two bacterial communities may have a protective effect on CVD [26]. *Bacteroides* can prevent atherosclerosis through a reduction in microbial LPS production [70]. *Phascolarctobacterium*, a genus of SCFA-producing bacteria, inhibits the growth of *Clostridium difficile* and holds promise as a therapeutic option for patients infected with *Clostridium difficile* [71]. However, in psoriatic patients, *Phascolarctobacterium* is considered a risk factor for CVD [72]. Further investigations are warranted to explore the impact of diverse microbial communities on the prognosis of HD patients.

Uremic toxins (TMAO, pCS, and IS) are associated with an increased CVD risk. Several studies have demonstrated that elevated TMAO levels represent an independent and significant risk factor for cardiovascular events in HD patients [73,74,75]. TMAO is associated with the progression of atherosclerosis, and its mechanisms include affecting cholesterol metabolism, promoting thrombosis, activating inflammation, and damaging endothelial cells [76,77]. TMAO increases the expression of receptor cluster of differentiation (CD) 36 and scavenger receptor A on macrophages, thereby inhibiting reverse cholesterol transport (RCT) and promoting the formation of foam cells [78]. TMAO induces mitogen-activated protein kinase and NF-κB signaling, thereby promoting vascular inflammation [79,80]. TMAO induces inflammation and endothelial dysfunction by activating the ROS-TXNIP-NLRP3 inflammasome pathway [81]. Moreover, elevated TMAO levels can upregulate the expression of thrombin, adenosine diphosphate (ADP), and collagen and trigger intracellular calcium release and platelet hyperreactivity, thereby contributing to the modulation of platelet function and thrombosis in vivo [82]. Recent studies also suggest that TMAO is closely associated with increased arterial stiffness and vascular calcification in HD patients [83,84]. An increase in arterial stiffness may lead to increased blood pressure and increased heart burden. Vascular calcification may cause the vessel wall to become fragile, increasing the risk of CVD. Cardiovascular calcification (CVC) is a well-known cardiovascular risk factor in HD patients [65].

Unlike TMAO, the protein-bound molecules pCS and IS are challenging to remove through conventional dialysis. The pathophysiological mechanisms associated with cardiovascular injury from pCS and IS include the induction of endothelial dysfunction, inflammatory responses, and oxidative stress [85]. Clinical studies have revealed a close association between these metabolites and the overall mortality and cardiovascular event mortality of HD patients [86,87,88,89,90]. These studies provide strong evidence that abnormal changes in metabolites may have a significant impact on the prognosis of HD patients. However, not all researchers have observed this significant correlation [91,92,93]. Therefore, further research is needed to determine the relationship between these metabolites and the prognosis of HD patients and whether changes in these metabolites can serve as biomarkers or targets for HD treatment. 

## 4. The Gut Microbiota and Infections

Infections are the second leading cause of death among ESRD patients, and sepsis accounts for more than 75% of all infection-related deaths, posing a serious threat to their lives and health [94]. Owing to impaired immune system function, these patients are more susceptible to infections by various pathogens, leading to infections and sepsis. In a 7-year follow-up of 4005 HD patients, 11.7% of HD patients had at least one episode of sepsis [95]. The mortality rate for sepsis in HD patients is significantly greater, ranging from 100–300 times greater than that of the general population [11]. The presence of an arteriovenous fistula/graft or a dialysis catheter, older age, malnutrition, diabetes, and the frequency of dialysis are predisposing factors for infections [96]. The pathogenic microorganisms that cause sepsis in HD patients are mainly Gram-positive bacteria, which include *Staphylococcus aureus* (*S. aureus*), especially methicillin-resistant *S. aureus* (MRSA). In addition, other staphylococci, including *S. epidermidis* and coagulase-negative *Staphylococcus* (CNS), are also common pathogens [97]. According to current research, the *S. aureus* found in the blood of HD patients may originate from the nasal microbiome [98]. 

Uremia can induce immune suppression, destroy the function of immune cells such as neutrophils, and reduce the host’s ability to defend against bacterial infections, thereby increasing the risk of infections for the host [99]. Studies have shown that pCS can inhibit T helper type 1 (TH1) cellular immune responses, which may lead to immune dysfunction in HD patients [100]. Clinical data also indicate that an elevated free pCS concentration in HD patients increases the risk of infection-related hospitalization (IH) [101,102]. Additionally, the translocation of the gut microbiota may also play a role in the development and progression of infections. These common microorganisms found in the human intestine, such as *Escherichia coli*, *Enterobacter*, and *Klebsiella*, are found in the blood of ESRD patients [97]. Polysaccharide A produced by *Bacteroides fragilis* serves as a crucial immune modulator capable of initiating T-cell-dependent immune response activation [103]. Research findings indicate a significant increase in *Bacteroides fragilis* among HD patients compared with healthy subjects [104]. It has been reported that *Bacteroides fragilis* can cause clinical infections such as anaerobic clavicular osteomyelitis and infections of the urinary tract [105,106].

HD patients are particularly susceptible to infections, and as a result, physicians often resort to antibiotic therapy to cure infections. However, prolonged or inappropriate use of antibiotics can lead to antibiotic resistance (ABR) [107,108,109]. Compared with healthy individuals, HD patients have an increased number of genes encoding erythromycin-resistant methylase [104]. The emergence of multidrug-resistant microorganisms (MDROs), such as *vancomycin-resistant enterococci* (VRE) and MRSA, among HD patients further complicates individual treatment and presents a significant public health challenge for healthcare facilities [110]. Therefore, when caring for HD patients, antibiotics must be used with caution. 

## 5. The Gut Microbiota and Anemia

Anemia is a common complication in HD patients. According to data from the China Dialysis Outcomes and Practice Patterns Study (DOPPS), 21% of patients receiving HD treatment in China had hemoglobin levels less than 9 g/dL [111]. These data highlight the widespread prevalence of anemia among dialysis patients in China, which significantly impacts their quality of life and prognosis. Notably, the corresponding figures in Japan and North America are 10% and 3%, respectively [111]. This may be related to differences in the level of development of dialysis treatment, medical resource allocation, and patient education in various countries.

There are many causes of anemia in HD patients, including altered iron homeostasis, erythropoietin (EPO) deficiency, hyperparathyroidism, and chronic inflammation [112]. Several in vitro experiments have shown that IS can inhibit the generation of EPO through a hypoxia-inducible factor (HIF)-dependent oxygen-sensing mechanism [113,114,115]. HIF is a transcription factor that is activated in low-oxygen environments and can bind to the promoter of the EPO gene, thereby regulating EPO generation [116]. Additionally, IS can induce suicidal erythrocyte death or eryptosis, both of which are associated with a shortened lifespan of erythrocytes [117]. However, no significant correlation between IS or pCS and anemia has been reported in clinical studies of HD patients [112,118]. The significant difference between the in vivo and in vitro experiments may be due to the significant differences in the experimental environments. The concentration of uremic toxins in in vitro experiments may be relatively high. Therefore, it is essential to fully consider this possibility when designing experiments to ensure the accuracy and reliability of the experimental results [112].

Iron supplementation and EPO-stimulating agents (ESAs) are commonly used in HD patients. However, EPO hyporesponsiveness (EH) occurs in 10% of patients treated with ESAs [119,120], which may be related to iron metabolism disorders and EPO receptor dysfunction [121]. In a study on the responsiveness to EPO treatment, researchers reported that nine bacteria in HD patients had predictive value for EH, with *Neisseria* showing the highest predictive value [122]. The authors also reported that the majority of enzymes related to butyrate synthesis were significantly enriched in HD patients with a good EH response, which may contribute to improving anemia [122]. A recent study has shown that supplementing dietary fiber (DF) can significantly improve anemia in HD patients. There are likely multiple mechanisms by which supplementing DF can improve renal anemia, one of which may be the increased production of butyrate and butyrate-producing bacteria (such as *Bifidobacterium*, *Lactobacillus*, and *Lactobacillaceae*) [121]. However, this discovery provides only a preliminary indication of this possibility. To ensure that this discovery can truly benefit HD patients, it is necessary to conduct multicenter, large-sample, and long-term clinical studies to comprehensively evaluate its clinical value.

## 6. The Gut Microbiota and Malnutrition

HD patients are at high risk of malnutrition due to impaired kidney function, which makes them prone to sarcopenia and protein-energy wasting (PEW). Under both of these conditions, the loss of muscle mass plays a crucial role in pathogenesis [123]. However, the concept of sarcopenia is no longer limited to a decrease in muscle mass but also includes the loss of muscle strength [124]. Protein loss during dialysis, reduced physical activity, chronic inflammation, etc., can all contribute to the development of sarcopenia [125]. The gut–muscle axis suggests that the gut microbiota plays a crucial role in maintaining skeletal muscle homeostasis [126]. A recent study demonstrated a decrease in gut microbiota diversity and alterations in microbial structure in HD patients with sarcopenia [127]. However, there have been few studies on HD patients, and most of them were observational studies with small sample sizes, which cannot prove a causal relationship between the gut microbiota and sarcopenia. Tang et al. reported significant reductions in muscle function and mass in mice colonized with gut microbiota from HD patients with sarcopenia, accompanied by a decrease in the abundance of *Akkermansia*, a producer of SCFAs [128]. Notably, SCFAs have been proven by multiple studies to have a positive effect on skeletal muscle mass [129,130]. Additionally, a study revealed that IS may cause metabolic disorders, further leading to impaired mitochondrial function [131]. Mitochondria are energy factories in cells and are crucial for the growth and maintenance of muscles. The impairment of mitochondrial function may ultimately result in a reduction in muscle mass [131]. Additionally, IS may also induce myotube atrophy in C2C12 cells by increasing oxidative stress and activating mitogen-activated protein kinases (MAPKs) [132]. However, many aspects of the pathogenesis of sarcopenia remain unknown, and future research will continue to explore these areas to provide more effective treatment options for HD patients.

Similarly, PEW is a disease related to malnutrition that primarily occurs in people who are unable to obtain sufficient food or nutrients, leading to severe deficiencies in body protein and energy [133]. The prevalence of PEW in HD patients is 30% to 75% [134]. Decreased nutrient intake, systemic inflammation, and inadequate dialysis are associated with the development of PEW [135,136]. The abundance of the butyric-acid-producing bacteria *Faecalibacterium prausnitzii* and *Roseburia* was reportedly reduced in HD patients with PEW [135,137]. The gut microbiota may also be useful for predicting PEW in HD patients [138]. The researchers reported a positive correlation between the levels of Actinobacteria and *Bifidobacteriaceae* and PEW indicators such as serum albumin levels, lean tissue mass (LTM), and the lean tissue index (LTI) [138]. The level of TMAO was significantly greater in HD patients with PEW [139]. These findings suggest that TMAO may play an important role in this disease process. Further research revealed that circulating TMAO levels are significantly associated with the incidence of PEW in HD patients [139], which means that an increase in TMAO levels may increase the risk of PEW. To better understand this phenomenon, it is necessary to conduct in-depth research on the causal relationship between TMAO and PEW.

## 7. The Gut Microbiota and Depression and Cognitive Impairment

The gut–brain axis refers to a bidirectional communication system in which the gut microbiota and the brain influence each other through neural, metabolic, and immune pathways [140,141]. This bidirectional signal transduction mechanism allows the gut–brain axis to play an important role in regulating mood and cognitive function.

Depression and cognitive impairment, which are common central nervous system (CNS) disorders of HD patients, not only have a negative impact on individuals but also impose a significant burden on society and the economy. According to survey data, the overall prevalence of depression among HD patients is 20–47% [142]. Depression is associated with a variety of factors, including a history of cerebrovascular disease, autonomic dysfunction, malnutrition, and inflammation [142]. Previous studies have shown that the levels of inflammatory markers such as IL-1, IL-6, and TNF-α are significantly elevated in HD patients with depression, suggesting that inflammation plays an important role in the pathogenesis of depression [142,143]. The uremic toxin IS levels seem to have a complex relationship with depression, with different outcomes in preclinical and clinical models. An experiment on mice with unilateral kidney removal after intraperitoneal injection of IS revealed that the levels of IS in their blood, prefrontal cortical tissues, and cerebrospinal fluid were elevated. Moreover, the mice were observed to exhibit behavioral symptoms of emotional disturbances such as anxiety, depression, and cognitive impairment [144]. This may be related to the pro-inflammatory effect of IS on astrocytes [145]. Interestingly, a study of the relationship between depression and IS found that the occurrence of depression was significantly and independently associated with lower total IS levels [146]. This phenomenon may be related to a reduction in tryptophan intake by the patient, leading to a decrease in the synthesis of 5-hydroxy tryptamine (5-HT, serotonin), as well as the direct antidepressant effect of total IS in the serum [146]. However, further research is needed to elucidate the pathophysiological role of IS in depression.

Similarly, cognitive impairment is associated with poor clinical outcomes in HD patients, with an incidence rate of from 30% to 60%, which is twice that reported in the general population [147]. A previous study revealed that the number and diversity of the gut microbiota in HD patients with mild cognitive decline (MCD) are significantly lower than those in healthy individuals and individuals with normal cognitive function, suggesting that cognitive dysfunction in HD patients is significantly related to an abnormal gut microbiota [148]. In addition, the levels of the class *Coriobacteriia* and the genera *Tyzzerella 3*, *Blautia*, and *Lachnospira* were significantly lower in HD patients with MCD [148]. Among these groups of bacteria, the genus *Blautia* has the highest diagnostic value, but the specific mechanism of action is still unclear. The study also revealed that higher levels of IS are associated with cognitive impairment in patients with early-stage CKD and HD patients [149,150]. However, no association was observed between pCS and cognitive impairment. This may be due to the difference in the protein-binding ability of IS and pCS. Specifically, IS has a stronger protein-binding ability than pCS, leading to a reduction in HD clearance and an increase in free IS levels. The specific mechanisms by which IS contributes to cognitive impairment may be related to its neurotoxicity. IS can induce oxidative stress and inflammation in astrocytes by activating NF-κB and aryl hydrocarbon receptors (AHRs), thereby disrupting the stability of the CNS and causing brain dysfunction [145].

## 8. Strategies to Attenuate Gut Microbiota Dysbiosis in HD

In recent years, research on probiotics, prebiotics, and synbiotics in the field of human health has received considerable attention. These three dietary supplements are believed to have significant effects on the balance of the gut microbiota and human health. In evaluating the effects of these supplements on diseases, most research currently uses indicators such as endotoxins, uremic toxins, inflammation, and metabolic markers. Probiotics, as active microorganisms, can not only reduce inflammatory responses [151] but also positively impact glucose homeostasis, oxidative stress [152,153], kidney function [153], nutritional status [154], and quality-of-life indicators [155,156]. Moreover, probiotics have been shown to significantly reduce the production of uremic toxin precursors (such as phenol and p-cresol) [157] but have no significant positive effect on IS or pCS in HD patients [158,159] (Table 2).

Prebiotics are indigestible food components that are primarily found in dietary fiber. They selectively stimulate the metabolism and proliferation of bacteria in the colon, thereby regulating the microbial balance of the intestine [171]. In fourteen HD patients who were supplemented with curcumin for three months, curcumin significantly lowered plasma pCS levels [166]. Resistant starch (RS) is a prebiotic compound that promotes the proliferation of SCFA-producing groups (such as *Roseburia* and *Ruminococcus gauvreauii*) [167], increases the production of SCFAs, and alleviates inflammation and oxidative stress markers [161,162]. Esgalhado et al. and Sirich, Plummer, et al. reported that RS only reduced plasma IS levels, with no significant effect on pCS levels [160,161]. High-amylose resistant starch (HAM-RS2) can reduce serum creatinine and p-cresol levels but has no significant effect on IS levels [163]. However, the efficacy of RS has been confirmed in many but not all studies. RS does not seem to play a role in altering plasma TMAO levels, as it does not contribute to any significant changes in the concentration of this metabolite in the bloodstream [170].

Synbiotics are mixtures of prebiotics and probiotics. A trial investigating synbiotic ingestion for four weeks in HD patients revealed that such therapy altered the fecal microbiota (*Bifidobacterium* enrichment) and significantly increased the levels of acetic acid and butyric acid [169]. In 58 HD subjects, a 7-week synbiotic treatment decreased the serum IS, pCS, and urea concentrations [164]. Probiotic intervention has demonstrated potential benefits in reducing the levels of serum hs-CRP, IL6, and endotoxins [165]. Furthermore, the combined administration of synbiotics and probiotics for 12 weeks has been shown to ameliorate anemia in HD patients [168].

AST-120 is an oral spherical carbon-based adsorbent used to lower uremic toxin levels in CKD patients and delay the start of dialysis [172]. According to the latest clinical research results, AST-120 seems to have a positive effect on HD patients. In addition to reducing the concentrations of IS, pCS, and inflammatory factors, AST-120 was also found to ameliorate pruritus and endothelial dysfunction caused by uremia [173,174,175]. However, the effectiveness of AST-120 in the clinical treatment of CKD is controversial [172]. Therefore, before it can be applied to the clinical treatment of HD patients, its efficacy needs to be validated on a larger scale and in a broader range of populations to determine its exact effect.

Fecal microbiota transplantation (FMT) is a treatment that involves delivering the gut microbiota of a healthy person to patients to regulate and rebuild their gut microbiota. In recent years, FMT has shown promising prospects and potential for clinical application, especially in treating *Clostridium difficile* infection (rCDI) with good efficacy [176]. However, as far as we know, there are very few publicly available clinical data on the application of FMT in kidney disease patients. In a clinical case report, a membranous nephropathy patient who received FMT twice presented significant alleviation of diarrhea symptoms and improvements in renal function indicators [177]. In another case report, two patients with IgA nephropathy (IgAN) who received FMT treatment for 6–7 months presented positive results. After treatment, there was a marked improvement in gut dysbiosis and in kidney function [178]. According to one study, HD may have a negative impact on the effectiveness of FMT treatment. In an assessment of different FMT protocols for the treatment of rCDI, patients who had received HD had a greater proportion of failed outcomes after FMT treatment [179]. Therefore, much work is still needed before FMT can be used as an effective intervention method for managing gut dysbiosis in HD patients.

## 9. Conclusions

In general, HD restored the abundance of beneficial microbes but induced some potentially pathogenic bacteria. The gut microbiota can play a crucial role in post-HD complications, such as cardiovascular events, infections, anemia, nutritional complications, and CNS disorders, through metabolites and immune regulation. Studies aimed at restoring the appropriate gut microbiota composition and alleviating uremic toxins have been conducted. It seems that dietary interventions consisting of probiotics, prebiotics, and synbiotics are promising strategies. These dietary supplements have been shown to restore the gut microbiota composition, reduce IS and pCS levels, and reduce inflammatory marker levels. However, there is a lack of clear guidelines to inform HD patients when to take these dietary supplements, as well as the types and doses that must be taken, which can confuse clinicians when prescribing them for patients. All of these measures have limited effects. They do not fundamentally solve the problem of gut microbiota imbalance.

In addition, there are many limitations in the study of the gut microbiota and metabolites in HD patients. First, research on the gut microbiota and metabolites in HD patients is mostly based on single-center, small-sample studies at present. These studies largely ignore the influence of diet, dialysis duration, and drug intake on the gut microbiota and metabolites, leading to highly heterogeneous conclusions and difficulty in forming a unified scientific understanding. Second, most of the existing studies are observational studies and have not fully demonstrated the causal relationship between the gut microbiota and the health status of HD patients. In addition, the molecular mechanisms by which metabolites regulate HD complications remain unclear, which limits our understanding and means of preventing and treating HD complications. Finally, reliable evidence from the gut microbiota perspective to guide clinical management strategies is challenging to obtain because of the complex relationships between hosts and microorganisms, individual differences, and susceptibility to multiple factors. This requires a deeper understanding of the diversity and dynamics of the gut microbiota, as well as further research on the relationship between the gut microbiota and the health status of HD patients to reveal the underlying biological mechanisms. In the future, we look forward to gaining a deeper understanding of the gut microbiota and metabolites of HD patients through large-scale clinical trials, considering the effects of diet, dialysis duration, and drug intake, to provide a more comprehensive and scientific basis for clinical management strategies.

## Figures and Tables

**Table 1:** Alterations in the gut microbiota in HD patients.

Study	Year	Group	Methods, Samples	Main Findings	Conclusions
Vaziri et al. [18]	2013	HD (*n* = 24) HC (*n* = 12)	16S *rRNA* gene sequencing, stool	↑ Actinobacteria, Firmicutes (especially Clostridia), and Proteobacteria (primarily Gammaproteobacteria) (HD > HC) ↑ *Brachybacterium*, *Catenibacterium*, *Enterobacteriaceae*, *Halomonadaceae*, *Moraxellaceae*, *Nesterenkonia*, *Polyangiaceae*, *Pseudomonadaceae*, and *Thiothrix* (HD > HC)	The uremic state can alter the composition of the gut microbiota
K. Shi et al. [23]	2014	HD (*n* = 22) ND (*n* = 30) HC (*n* = 12)	16S *rRNA* gene sequencing, stool and blood	↑ Proteobacteria in the gut and blood (HD > ND > HC) ↑ *Bacteroides* in the gut and blood (HD and ND > HC) ↓ Firmicutes and Bacteroidetes in the gut (HD and ND < HC)	HD may exaggerate microinflammation in ESRD patients
J. Crespo-Salgado et al. [25]	2016	PD (*n* = 8) HD (*n* = 8) post-kidney transplant (*n* = 10) HC (*n* = 13)	16S *rRNA* gene sequencing, stool	↑ Bacteroidetes (HD > HC) ↑ Proteobacteria (PD > HD) ↓ Actinobacteria (PD < HC) ↑ *Enterobacteriaceae* (PD > HC) ↓ *Bifidobacteriaceae* (PD and post-kidney transplant < HC)	Children with ESRD have altered gut microbiota and increased serum uremic toxins derived from bacteria
Luo et al. [26]	2021	ND (*n* = 33), PD (*n* = 19) HD (*n* = 21) HC (*n* = 19)	16S *rRNA* gene sequencing, stool	↓ Bacteroidetes (HD < HC, ND, and PD) ↑ *Blautia* and *Dorea* (PD and HD > HC) ↑ *Akkermansia*, *Coprococcus*, *Acinetobacter*, *Proteus* and *Pseudomonas* (HD > ND) ↓ *Prevotella* and *Paraprevotella* (HD < ND) ↓ *Prevotella* (PD and HD < HC)	RRT alters the composition of the gut microbiota, and these alterations were pronounced in HD patients. HD restored the relative abundance of beneficial bacteria and induced some potential pathogenic bacteria
H. Y. Wu et al. [24]	2023	HD (*n* = 96) HC (*n* = 81)	16S *rRNA* gene sequencing, stool	↑ Firmicutes, Proteobacteria, *Escherichia*, *Streptococcus*, *Lactobacillus*, *Enterococcus*, *Staphylococcus*, *Klebsiella* (HD > HC) ↓ Bacteriodetes, *Bifidobacterium*, *Prevotella*, and *Bacteroides* (HD < HC)	There is a significant difference in the composition of the gut microbiota between HD patients and healthy subjects

↑, increase; ↓, decrease; HD, hemodialysis; PD, peritoneal dialysis; HC, healthy control; ND, nondialysis; RRT, renal replacement therapy.

**Table 2:** Summary of the impact of dietary supplements (probiotics, prebiotics, and synbiotics) in HD patients.

Reference	Year	Type of Study, Sample Size	Dietary Supplementation, Duration	Results	Conclusions
Hyun et al. [159]	2013	Single-center, prospective, nonrandomized, open-label trial, HD patients (*n* = 5), PD patients (*n* = 11)	Probiotic: *Lactobacillus casei*, *Lactobacillus plantarum*, *Lactobacillus acidophilus*, and *Lactobacillus delbrueckii* subsp. *Bulgaricus*, 12 weeks	There were no significant changes in the levels of the uremic toxins pCS and IS after treatment with probiotics	Probiotics had no positive effect on uremic toxin levels in pediatric dialysis patients.
Sirich, Tammy L et al. [160]	2014	Randomized, single-blinded, prospective trial, HD patients (*n* = 40)	Prebiotics: fiber sachets contained 15 g of high-amylose corn starch, 6 weeks	Reduced the unbound, free plasma level of IS	Increasing dietary fiber can lower the plasma levels of uremic toxins
Soleimani et al. [152]	2017	Randomized, double-blind, placebo-controlled, clinical trial, diabetic HD patients (*n* = 60)	Probiotic: contained *L. acidophilus*, *L. casei*, and *B. bifidum*, 12 weeks	Decreased in FPG, insulin, HOMA-IR, HbA1c, hs-CRP, MDA, SGA scores, and TIBC; Increased plasma TAC and QUICKI	Probiotics had benefits on insulin metabolism parameters, lipid profiles, biomarkers of inflammation, and oxidative stress in diabetic HD patients
Borges et al. [158]	2018	Randomized, double-blind, placebo-controlled trial, HD patients (*n* = 46)	Probiotic: *Streptococcus thermophilus*, *Lactobacillus acidophilus*, and *Bifidobacteria longum*, 3 months	Increased serum urea, potassium, and IS; Reduced the fecal pH	Probiotic supplementation failed to reduce uremic toxins and inflammatory markers
Eidi et al. [157]	2018	Randomized, double-blind, clinical trial, HD patients (*n* = 42)	Probiotic: *Lactobacillus rhamnosus*, 4 weeks	Decreased in p-cresol and phenol levels	Probiotics may be a promising treatment in HD patients by reducing the serum phenolic uremic toxins
Esgalhado et al. [161]	2018	Randomized, controlled, pilot trial, HD patients (*n* = 40)	Prebiotics: resistant starch, 4 weeks	Reduced plasma levels of IL-6 and IS	RS supplementation may be a promising nutritional strategy to improve inflammation, oxidative stress, and to reduce IS plasma levels in HD patients
Tayebi Khosroshahi et al. [162]	2018	Randomized clinical trial, HD patients (*n* = 46)	Prebiotics: HAM-RS2 20 g/d (the first 4 weeks) and 25 g/d (the second 4 weeks)	Reduced serum levels of TNF-α, IL-6, and MDA, urea, and creatinine	Administration of HAM-RS2 for eight weeks significantly reduced levels of inflammatory and oxidative markers in HD patients
Khosroshahi, Hamid Tayebi et al. [163]	2019	Randomized, controlled trial, HD patients (*n* = 50)	Prebiotics: HAM-RS2, 8 weeks	Reduced serum levels of p-cresol, creatinine, and uric acid but did not lower the level of IS.	Administration of fermentable high-fiber diet as HAM-RS2 can reduce some indicators related to kidney function and affect certain metabolic products produced in the gut
Lopes et al. [164]	2019	Randomized, single-blind, controlled study, HD patients (*n* = 58)	Synbiotic: pasteurized milk and *B. longum* BL-G301, 7 weeks	Decreased serum pCS, IS, and urea concentration	Synbiotic reduced the pCS and IS serum uremic toxins and urea in HD patients
Haghighat, Mohammadshahi, Shayanpour, and Haghighizadeh [165]	2020	Randomized, double-blinded, controlled clinical trial, HD patients (*n* = 75)	Synbiotic: *Lactobacillus acidophilus* T16, *Bifidobacterium bifidum* BIA 6, *Bifidobacterium lactis* BIA6, and *B. longum* LAF-5 and prebiotics (contained fructooligosaccharides (FOS), galactooligosaccharides (GOS), and inulin, 12 weeks	Decreased hs-CRP, anti-HSP70, and endotoxin	Synbiotics were more effective than probiotics for improvement in inflammatory markers, endotoxin, and anti-HSP70 serum levels
Belova et al. [155]	2021	Randomized, parallel-group, controlled clinical trial, HD patients (*n* = 62)	Probiotic component from immobilized multistrain synbiotic LB-complex L	Restored 56% of the patients’ microbiocenosis; Decreased CRP and ESR and improved quality of life	Adding probiotic in the diet can improve dysbiosis and quality of life
Salarolli et al. [166]	2021	Randomized, double-blind, controlled pilot study, HD patients (*n* = 28)	Prebiotics: curcumin, 12 weeks	Reduced the level of pCS but did not lower the level of IS	Curcumin could reduce pCS plasma levels of HD patients
Kemp et al. [167]	2021	Randomized, double-blind, placebo-controlled trial, HD patients (*n* = 20)	Prebiotics: RS2, 4 weeks	Increased *Oscillosperaceae*, *Roseburia*, *Ruminococcus gauvreauii*; Decreased *Ruminococcus champanellens*, *Dialister*, *Coprococcus*	RS2 can effectively alter SCFAs producers, may be a good nutritional strategy for HD patients
Haghighat et al. [168]	2021	Randomized, double-blind, placebo-controlled trial, HD patients (*n* = 75)	Synbiotic: *Lactobacillus acidophilus* strain T16, *Bifidobacterium bifidum* strain BIA-6, *Bifidobacterium lactis* strain BIA-7, *B. longum* strain BIA-8 and prebiotics includes fructooligosaccharides (FOS), galactooligosaccharides (GOS), inulin, 12 weeks	Increased serum Hb level and decreased BDI and BAI score but did not change the HRQoL score	Synbiotic and probiotic supplements have played a positive role in improving mental health and alleviating anemia. However, there has been no significant impact on improving quality of life of HD patients
Miyoshi, Kadoguchi, Usami, and Hori [169]	2021	Two-arm, nonrandomized, controlled trial, HD patients (*n* = 11)	Synbiotics: colony-forming units of *B. longum* BB536 and PHGG	Improved the individual stool form and abdominal bloating and increased *Bifidobacterium*, *Bacteroides*, and *Clostridium* (IV, IX) and butyric acid concentration	The improvement in the stool form by synbiotics helped to relieve constipation, thus improving the quality of life of HD patients
Shamanadze et al. [156]	2022	Single-center, cohort-prospective study, HD patients (*n* = 40)	Probiotic: *L. acidophilus* KB27, *B. longum* KB31, and *S. thermophilus* KB19. 34, 2 weeks	Improved gut microbiota and gastrointestinal complaints, such as meteorism, diarrhea, and fulness	Probiotics had beneficial effects on some gastrointestinal complaints and improved the quality of life in HD patients
M. Yamamoto et al. [154]	2022	Retrospective study, HD patients (*n* = 37)	Probiotics: *Clostridium* *butyricum*, 3 times per day for 2 years	Increased gut microbiota abundant and the albumin level, transferrin level, and E/T ratio; Decreased the IL-6 level	*Clostridium butyricum* positively affects the gut microbiota and nutritional and immunological statuses
Choi et al. [151]	2022	Not a randomized, controlled or crossover study, HD (*n* = 22)	Probiotics: *Bifidobacterium bifidum* BGN4 and *B. longum* BORI, twice per day for 3 months	Fecal SCFAs and anti-inflammatory in the percentage of CD4^+^ CD25^+^ Tregs increased; Serum levels of calprotectin and IL-6 upon LPS stimulation, and CD14^+^ CD16^+^ proinflammatory monocytes decreased	Probiotic could reduce systemic inflammatory responses
Kemp et al. [170]	2022	Randomized, double-blind, placebo-controlled trial, HD patients (*n* = 25)	Prebiotics: RS for 4 weeks	RS supplementation did not change plasma TMAO, choline, betaine, or fecal taxa potentially linked to TMAO	RS does not seem to modify the TMA-associated bacterial taxa, precursors of TMAO
Y. Zhang et al. [153]	2023	Placebo-controlled clinical trial, diabetic HD (*n* = 86)	Probiotic: *Lactobacillus acidophilus*, *Lactobacillus casei*, and *Bifidobacterium*, for 12 weeks	Serum ghrelin levels, the nutrient intake, and glutathione increased; Serum adiponectin creatinine, FBG, uPCR, HOMA-IR, MDA, CRP, and TNF-α decreased	Probiotics have a positive effect on blood sugar control, insulin resistance, and renal function

HD, hemodialysis; PD, peritoneal dialysis; IS, indoxyl sulfate; pCS, p-cresyl sulfate; FPG, fasting plasma glucose; HOMA-IR, homeostasis model of assessment-estimated insulin resistance; hs-CRP, high-sensitivity C-reactive protein; MDA, malondialdehyde; SGA, subjective global assessment; TIBC, total iron binding capacity; QUICKI, quantitative insulin sensitivity check index; RS, resistant starch; HAM-RS2, high-amylose maize resistant starch; *B. longum*, *Bifidobacterium longum*; ESR, erythrocyte sedimentation rate; RS2, resistant starch type-2; SCFAs, short-chain fatty acids; PHGG, partially hydrolyzed guar gum; BDI, Beck Depression Index; BAI, Beck Anxiety Index; HRQoL, health-related quality of life; E/T ratio, effector cell/target cell ratio; IL-6, interleukin-6; LPS, lipopolysaccharide; TMAO, trimethylamine N-oxide; TMA, trimethylamine; FBG, fasting blood glucose; uPCR, urine protein–creatinine ratio; TNF-α, tumor necrosis factor-α.
